# A Critical Role for PDGFRα Signaling in Medial Nasal Process Development

**DOI:** 10.1371/journal.pgen.1003851

**Published:** 2013-09-26

**Authors:** Fenglei He, Philippe Soriano

**Affiliations:** Department of Developmental and Regenerative Biology, Icahn School of Medicine at Mount Sinai, New York, New York, United States of America; California Institute of Technology, United States of America

## Abstract

The primitive face is composed of neural crest cell (NCC) derived prominences. The medial nasal processes (MNP) give rise to the upper lip and vomeronasal organ, and are essential for normal craniofacial development, but the mechanism of MNP development remains largely unknown. PDGFRα signaling is known to be critical for NCC development and craniofacial morphogenesis. In this study, we show that PDGFRα is required for MNP development by maintaining the migration of progenitor neural crest cells (NCCs) and the proliferation of MNP cells. Further investigations reveal that PI3K/Akt and Rac1 signaling mediate PDGFRα function during MNP development. We thus establish PDGFRα as a novel regulator of MNP development and elucidate the roles of its downstream signaling pathways at cellular and molecular levels.

## Author Summary

Craniofacial anomalies, including cleft lip and palate, are frequent birth defects. Although these are often associated with defects in neural crest development, the more severe phenotypic manifestations of midline defects is facial clefting, which is poorly understood. In this work, we show that the facial clefting phenotype of PDGFRα mutants is not associated with a defect in neural crest cell specification but rather a subsequent defect in the medial nasal process (MNP), a facial primordium derived from the frontonasal prominence. We further show that this defect is associated with alterations in both cell proliferation and cell migration, and that PI3K and Rac1 signaling are essential to maintain a normal level of cell proliferation. Last, we provide evidence that Rac1 regulates cell migration at the level of cell motility as well as chemotaxis under the regulation of PDGFRα. We thus establish PDGFRα as a novel regulator of MNP development and elucidate the roles of its downstream signaling pathways at cellular and molecular levels.

## Introduction

Neural crest cells (NCCs) are a transient and multipotent cell population unique to vertebrates. During development, NCCs give rise to a broad variety of cell types, which contribute to the formation of the peripheral nervous system, cardiac outflow tract, pigment cells, and the majority of craniofacial bones and cartilages [1]–[4]. Alterations of cranial NCC (cNCC) development often lead to craniofacial malformations, one of the most prevalent birth defects [5]. These facts underscore the significance of understanding the mechanisms regulating NCCs during craniofacial morphogenesis.

At the onset of craniofacial development, the facial primordium is composed of five prominences surrounding the stomodeum: the frontonasal prominence (FNP) at the rostral region, two paired maxillary processes in the middle, and a pair of mandibular processes at the caudal end [6], [7]. These primordia are populated predominantly by NCC derived cells, surrounding a mesodermal core and covered by the overlying ectoderm. The ectoderm then thickens and invaginates to form two bilateral nasal placodes, dividing the FNP into the medial nasal process (MNP) and a pair of lateral nasal processes (LNP). The MNP and bilateral maxillary processes contribute together to form the upper lip [8]. In mammals, the MNP further develops into the philtrum and the nasal septum, which later forms the nasal cartilage and bone [9]. Disruption of the MNP usually causes a variety of craniofacial defects, ranging from mild hypoplasia of the nasal bones to complete midfacial clefting. A number of genes regulate maxilla and mandible development, but it remains largely unknown how MNP development is controlled at the molecular and cellular level. Vital dye labeling studies reveal that the NCCs giving rise to different facial prominences share distinct origins along the rostral-caudal axis: NCCs from the diencephalon and anterior mesencephalon give rise to the MNP and LNP, while those originating from the posterior mesencephalon and hindbrain give rise to the maxilla and mandible [10], [11]. These results suggest that the MNP and other prominences may be regulated through different mechanisms.

Multiple genetic factors have been implicated in cranial NCC (cNCC) development. Among these, growth factor signaling pathways are essential for induction, proliferation, survival and migration [12]–[14]. BMP, FGF and Wnt signaling together mediate induction of cNCCs from neural ectoderm [13], [15]. cNCC proliferation and survival are under the control of BMP, FGF and TGFβ signaling, and migration of the cNCCs at the caudal level is regulated by BMP, Wnt, Semaphorin and Ephrin signaling [13]. Growth factors act via binding and activation of their cell surface receptors, which in turn engage multiple intracellular signaling pathways. It remains to be elucidated how these intracellular signaling pathways mediate the receptors' function, especially in developmental contexts.

Platelet Derived Growth Factor (PDGF) signaling plays essential roles in development and disease [16]–[19]. In mammals, PDGF signaling can be activated by four PDGF ligands (A, B, C and D) operating through two receptor tyrosine kinases, PDGFRα and β [17], [20]. Activation of PDGFRs leads to phosphorylation of intracellular tyrosines and docking of intracellular effectors, which in turn engage downstream signaling cascades including the MAPK, PI3K, PLCγ, STAT and Src pathways. Previous studies from our laboratory and others have shown that PDGFRα and its downstream signaling pathways are crucial for cardiac and cranial NCCs [21]–[24]. PDGFA/PDGFRα signaling has also been implicated in cell migration in zebrafish palatogenesis [25]. However, the precise mechanisms by which PDGFRα regulates cNCCs and MNP development still remain to be elucidated. To this end, we have carried out a detailed study of PDGFRα NCC conditional knockout embryos. Our work reveals novel roles of PDGFRα in regulating NCC migration, and for PDGFRα engaged PI3K signaling in MNP neural crest cell proliferation. Moreover, we show that Rac1 and PI3K signaling mediate these processes under the control of PDGFRα.

## Results

### Loss of PDGFRα leads to facial clefting due to loss of MNP derived structures

**Figure 1:**
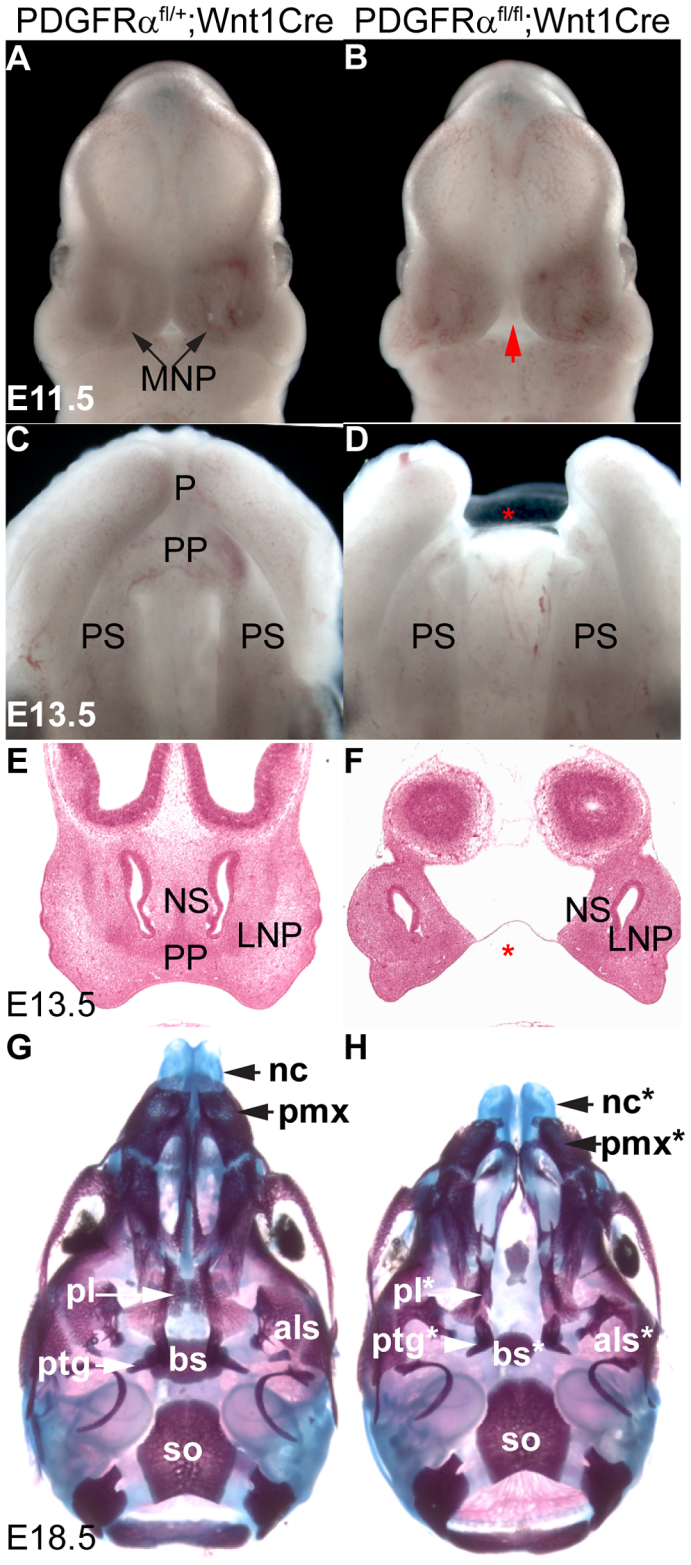
Tissue specific inactivation of PDGFRα from NCCs causes facial clefting and absence of MNP derived structures. (A, B) Frontal view of PDGFRα^fl/+^; Wnt1Cre (A) and PDGFRα^fl/fl^; Wnt1Cre embryo (B) at E11.5. An obvious gap was observed between MNPs in PDGFRα^fl/fl^; Wnt1Cre embryo (arrow in B). (C, D) Ventral view of PDGFRα^fl/+^; Wnt1Cre (C) and PDGFRα^fl/fl^; Wnt1Cre embryo (D) at E13.5. The PDGFRα^fl/fl^; Wnt1Cre embryo shows cleft lip and lacks of philtrum and primary palate (asterisk in D). The mandible has been removed for visualization. (E, F) Histological coronal sections of PDGFRα^fl/+^; Wnt1Cre (E) and PDGFRα^fl/fl^; Wnt1Cre embryo (F) at E13.5. PDGFRα^fl/fl^; Wnt1Cre embryo shows a cleft of the nasal septum and lacks the primary palate (asterisk in F). (G, H) Ventral view of skeletal preparation of PDGFRα^fl/+^; Wnt1Cre (G) and PDGFRα^fl/fl^; Wnt1Cre embryo (H) at E18.5. Note the shortening and clefting of the nasal cartilage in the PDGFRα^fl/fl^; Wnt1Cre embryo. Asterisks refer to defective bones or cartilages. Als, alisphenoid; bs, basisphenoid; LNP, lateral nasal process; MNP, medial nasal process; nc, nasal cartilage; NS, nasal septum; P, philtrum; pl, palatine; pmx, premaxilla; PP, primary palate; PS, palatal shelf; ptg, pterygoid.

To understand how PDGFRα and its downstream signaling pathways regulate NCC development and craniofacial morphogenesis, we generated NCC-specific PDGFRα conditional knockout (cKO) embryos by intercrossing PDGFRα^fl/fl^ and Wnt1Cre transgenic mice [21], [26]. Morphological differences first became visible at E11.5, as the medial nasal processes (MNP) of cKO littermates remained separated by an obvious gap relative to the control embryos (Fig. 1A, B). By E13.5, cKO embryos lacked the philtrum and the primary palate (Fig. 1C, D), both of which are derived from the MNP. This observation was confirmed by histological analysis that showed the absence of the primary palate and philtrum in the cKO embryos (Fig. 1E, F). At E18.5, skeletal preparations showed that PDGFRα^fl/fl^; Wnt1 Cre embryos exhibited a significant cleft (6 out of 6) and shortening of nasal cartilage (88±4.8% relative to control) and premaxilla (81.2±2.0% relative to control) (Fig. 1G, H). Skeletal analysis of cKO mutants also revealed malformation of the neural crest derived basisphenoid, alisphenoid and pterygoid bones (Fig. 1G, H) [1], [27], [28]. Other cNCC derived structures, such as the mandible, developed normally in the cKO mutants. No cKO embryos survived past birth.

**Figure 2:**
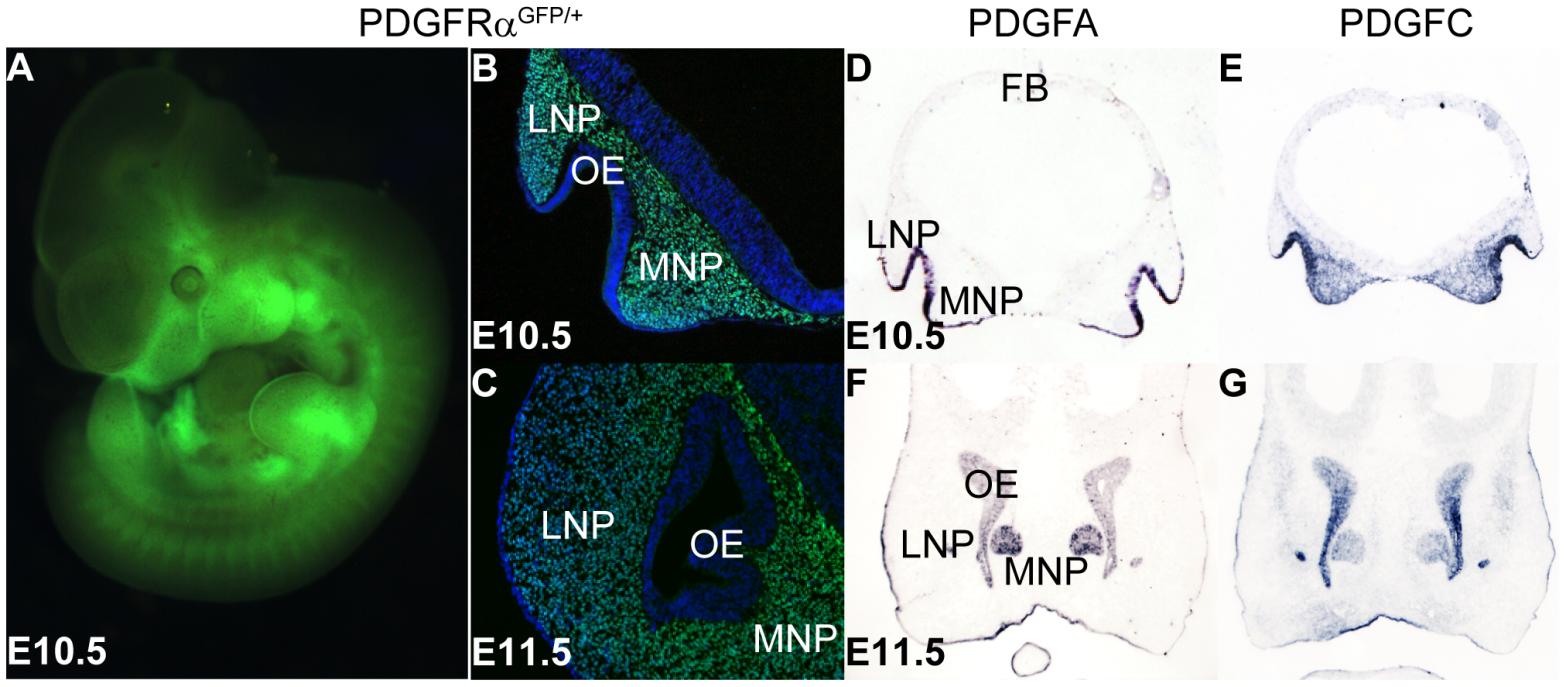
PDGFRα and its ligands PDGFA and PDGFC are expressed during midface development. (A) Whole mount view of PDGFRα expression, represented by H2B-GFP in the E10.5 PDGFRα^GFP/+^ knockin mouse embryo. (B, C) Coronal section shows PDGFRα expression in the mesenchyme of MNP and LNP at E10.5 and E11.5. E11.5 PDGFRα^GFP/+^ embryo shows high PDGFRα expression in the MNP mesenchyme, and a moderate level in the LNP. (D–G) In situ hybridization results show PDGFA and PDGFC are expressed in MNP and LNP at E10.5 and E11.5. At both stages, PDGFA is expressed in the epithelium of LNP and MNP, and the olfactory epithelium (D, F). PDGFC expression is detected in the epithelium, as well as in the LNP and MNP mesenchyme (E, G). LNP: Lateral Nasal Process; MNP: Medial Nasal Process; OE: Olfactory Epithelium.

To further understand how PDGFRα signals during MNP development, we analyzed the expression of PDGFRα and its ligands. Using PDGFRα^GFP/+^ knockin reporter mice [29], we observed broad GFP expression in the facial structures at E10.5 (Fig. 2A). Coronal sections of PDGFRα^GFP/+^ embryos at different stages revealed that PDGFRα is exclusively expressed in mesenchymal cells of the future facial structures, which are derived predominantly from cNCCs [1]. At E10.5, PDGFRα appears to be expressed equally in both the MNP and the LNP (Fig. 2B); however by E11.5, PDGFRα expression is reduced in the LNP relative to the MNP mesenchyme (Fig. 2C). PDGFA and PDGFC encode two endogenous ligands that bind specifically to PDGFRα, and inactivation of the two genes together recapitulates the PDGFRα null mutant phenotype [30]. In situ hybridization showed that these genes exhibit overlapping expression in the MNP: PDGFA is expressed in the MNP and LNP epithelium and PDGFC is expressed in both the epithelium and mesenchyme (Fig. 2D–G). The expression patterns of PDGFRα and its ligands indicate paracrine and possibly autocrine PDGFRα signaling during MNP development.

### PDGFRα regulates cell proliferation in the FNP and MNP through PI3K signaling

**Figure 3:**
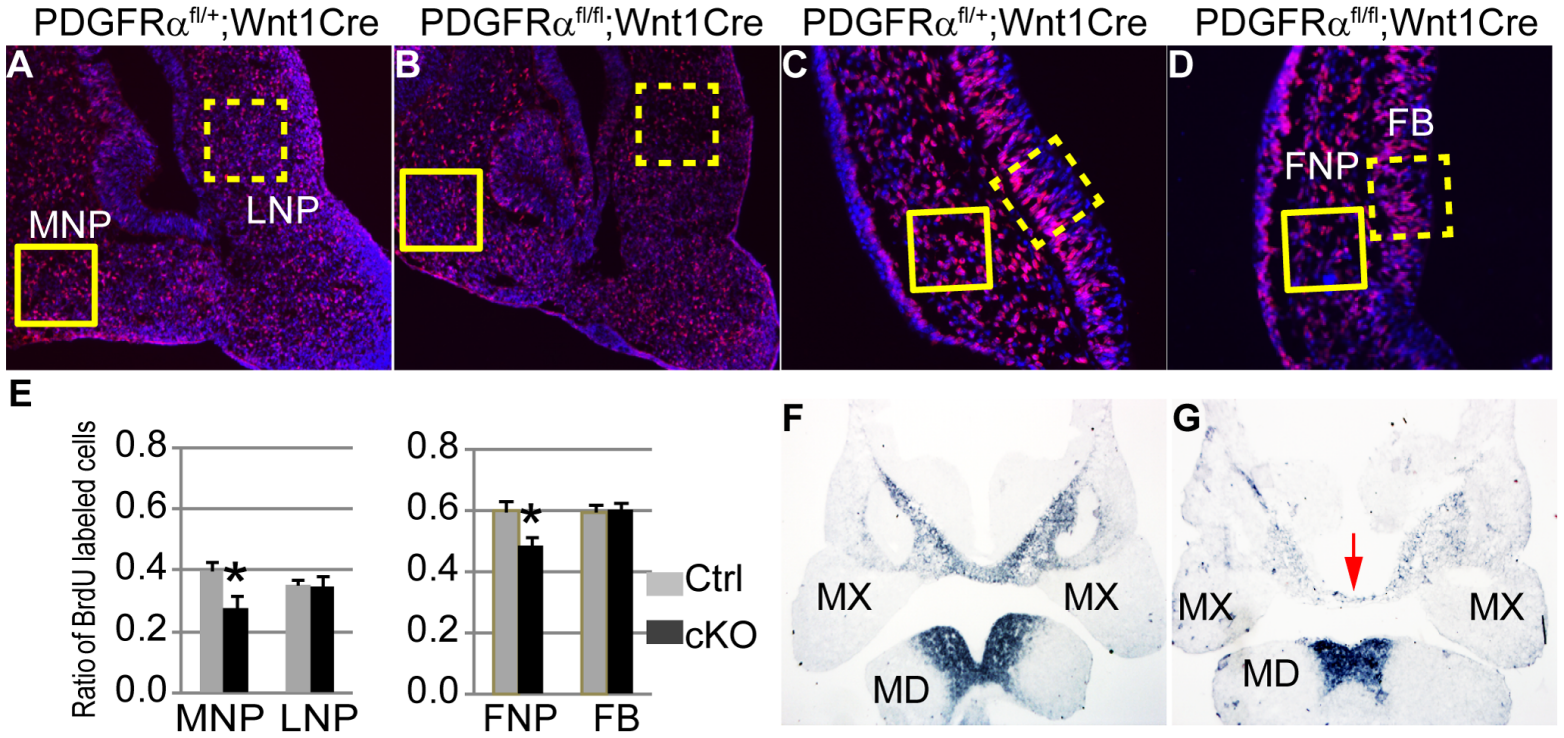
PDGFRα is essential to maintain cell proliferation and gene expression of MNP. (A–D) Coronal sections of BrdU (red) labeled PDGFRα^fl/+^; Wnt1Cre (A, C) and PDGFRα^fl/fl^; Wnt1Cre (B, D) embryos at E11.5 and E9.5. Nuclei are counterstained with DAPI (blue). (E) The cell proliferation rate is significantly decreased in the cKO MNP at E11.5 (PDGFRα^fl/fl^; Wnt1Cre), as compared to the Control (Ctrl; PDGFRα^fl/+^; Wnt1Cre). Control and cKO embryos exhibit comparable cell proliferation rates in the LNP. cKO embryos show decreased rate of cell proliferation in the FNP at E9.5, but a comparable rate in the FB relative to the control embryos. N = 9; asterisk, p<0.01. (F, G) Alx3 expression in PDGFRα^fl/+^; Wnt1Cre and PDGFRα^fl/fl^; Wnt1Cre embryos at E10.5. In the control embryo (F), Alx3 is expressed in the mesenchyme of medial nasal process and mandible at E10.5. Arrow points to the MNP. In the cKO embryo (G), Alx3 mRNA expression level is decreased specifically in the MNP mesenchyme, and remains comparable to the control in the mandible. FB, forebrain; FNP, frontonasal prominence; MD, mandible; MNP, medial nasal process; MX, maxilla; LNP, lateral nasal process.

To examine the role of PDGFRα in the MNP, we first analyzed cell proliferation and apoptosis in developing embryos. BrdU labeling revealed that in control embryos (n = 9), 40±3% of MNP mesenchymal cells and 35±2% of LNP mesenchymal cells were proliferating at E11.5. In cKO embryos, MNP mesenchymal cell proliferation was decreased by 30% relative to littermate controls, while LNP mesenchymal cell division was maintained a comparable level (Fig. 3A, B and E). No ectopic apoptosis was identified in cKO MNP cells (data not shown). Since the MNP and LNP are derived from the FNP during embryogenesis, we further traced these defects to the FNP. We found that at E9.5, cKO FNP mesenchymal cell proliferation was significantly downregulated by 19% (Fig. 3C–E), and no ectopic cell apoptosis was identified (data not shown). We also assayed expression of genes critical for MNP development in E10.5 embryos. Among them, Six1 and Alx4 expression remained unaltered in cKO and control embryos (Fig. S1), while Alx3 expression was downregulated in the cKO MNP mesenchyme, but not in the mandible (Fig. 3F, G). Because Alx3 is essential for MNP cell survival and no ectopic apoptosis was found in the cKO MNP (data not shown) [31], the reduction of Alx3 mRNA might be caused by a decrease in cell number in the cKO MNP. A similar change in gene expression has been observed in Pax6 mutant embryos, which showed a migration defect of MNP progenitor cells [32]. Together, these data suggest that PDGFRα regulates cell proliferation in the FNP and specifically in the MNP.

**Figure 4:**
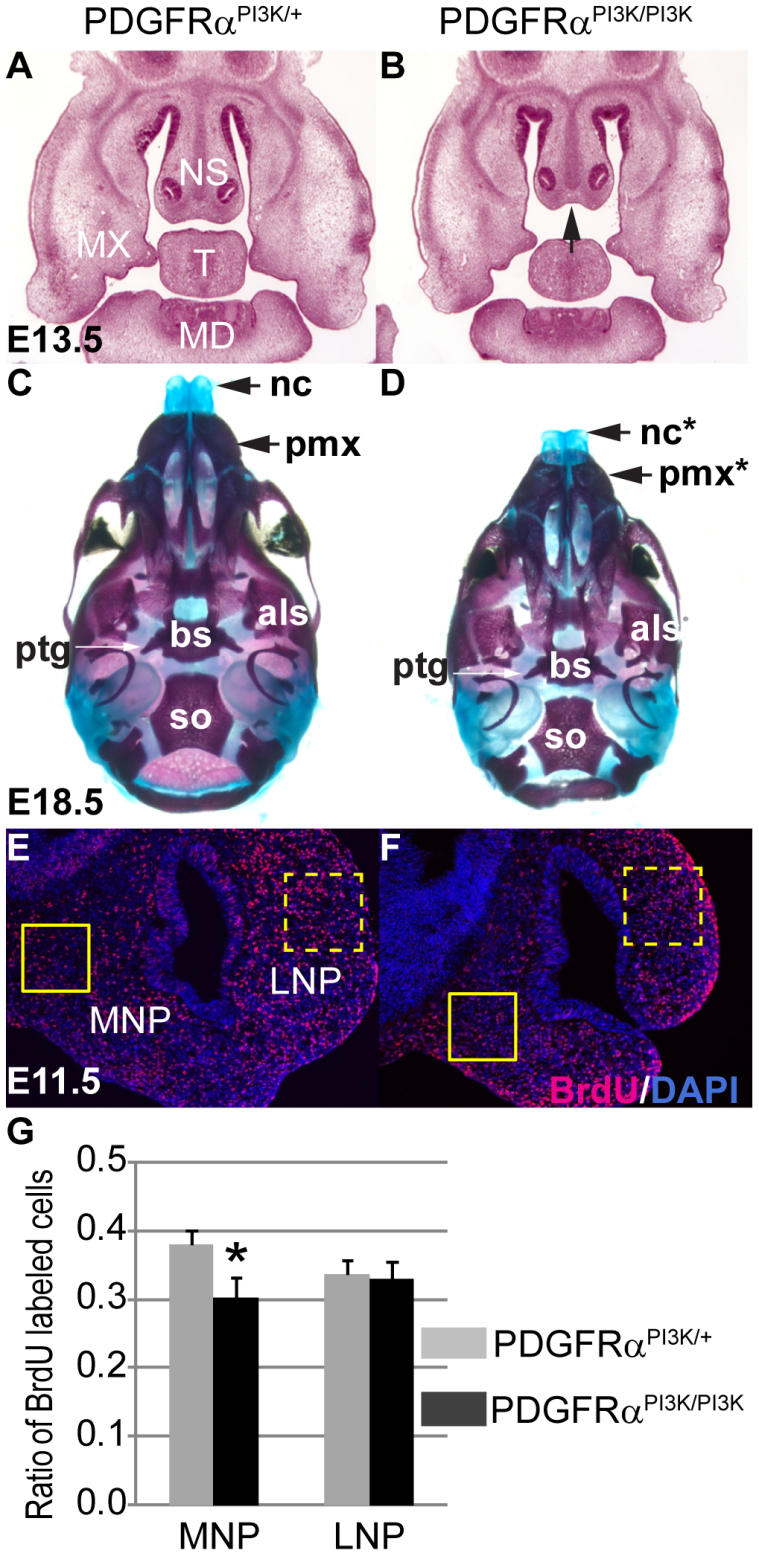
PI3K signaling mediates PDGFRα regulation on cell proliferation during MNP development. (A, B) Histological coronal sections of embryos at E13.5. PDGFRα^PI3K/PI3K^ embryo (B) exhibits a shortened nasal septum (arrow) and hypoplastic mandible and tongue, as compared to the PDGFRα^PI3K/+^ control (A). (C, D) PDGFRα^PI3K/PI3K^ E18.5 embryos develop hypoplastic nasal cartilages and shorter premaxilla bones than control (C). Asterisks refer to defective bones or cartilages. (E, F) At E11.5, BrdU labeling results show a decreased proliferation rate in PDGFRα^PI3K/PI3K^ MNP mesenchymal cells (F) as compared to the control (E). (G) The ratio of proliferating cells is significantly decreased in PDGFRα^PI3K/PI3K^ MNP mesenchyme when compared to that of PDGFRα^PI3K/+^. The LNP cell proliferation rate in PDGFRα^PI3K/PI3K^ is not affected. N = 9; asterisk, p<0.01. Als, alisphenoid; bs, basisphenoid; FB, forebrain; MD, mandible; MNP, medial nasal process; MX, maxilla; nc, nasal cartilage; NS, nasal septum; pmx, premaxilla; ptg, pterygoid; so, supraoccipital bone; T, tongue.

PDGFRα regulates cell fates and behaviors through a number of downstream signaling pathways. Previous studies in our laboratory showed that PI3K signaling is the major effector of PDGFRα in craniofacial development. Loss of PDGFRα-mediated PI3K signaling alone caused cleft palate with incomplete penetrance, but inactivation of PDGFRα together with PDGFRβ-mediated PI3K signaling caused facial clefting similar to PDGFRα null mutant embryos [24]. To examine the role of PI3K signaling in MNP development, we first analyzed the phenotype of PDGFRα^PI3K/PI3K^ mice. PDGFRα^PI3K/PI3K^ embryos exhibited a shortened nasal septum at E13.5 (Fig. 4A, B) and shortened nasal bones at E18.5 (Fig. 4C, D). The mutant nasal cartilage was 10% shorter than the heterozygous control and the premaxilla was 14% shorter than the control (n = 5, p<0.05). As with the cKO MNP, BrdU labeling results revealed that a decrease in cell proliferation in the MNP of PDGFRα^PI3K/PI3K^ embryos at E11.5 (Fig. 4E–G). To further substantiate these results, we extended these studies to Mouse Embryonic Palatal Mesenchymal cells (MEPMs). Although MNP cells would be the ideal material at this point, we were not able to maintain MNP cells in culture beyond passage 1. Similar to MNP mesenchymal cells, MEPMs originate from cNCCs, exhibit stable PDGFRα expression and response to PDGFA stimulation (Fig. S2) and are thus a robust tool to study PDGFRα function. PDGFA treatment significantly increased cell proliferation in WT MEPMs, but failed to do so in PDGFRα^PI3K/PI3K^ MEPMs (Fig. S3). Conversely, PDGFRα^PI3K/PI3K^ MEPMs exhibited a small decrease in cell proliferation (data not shown). Together these results indicate that PI3K signaling is essential for PDGFRα-regulated neural crest derived cell proliferation.

### Neural crest cells fail to populate the frontonasal area efficiently

**Figure 5:**
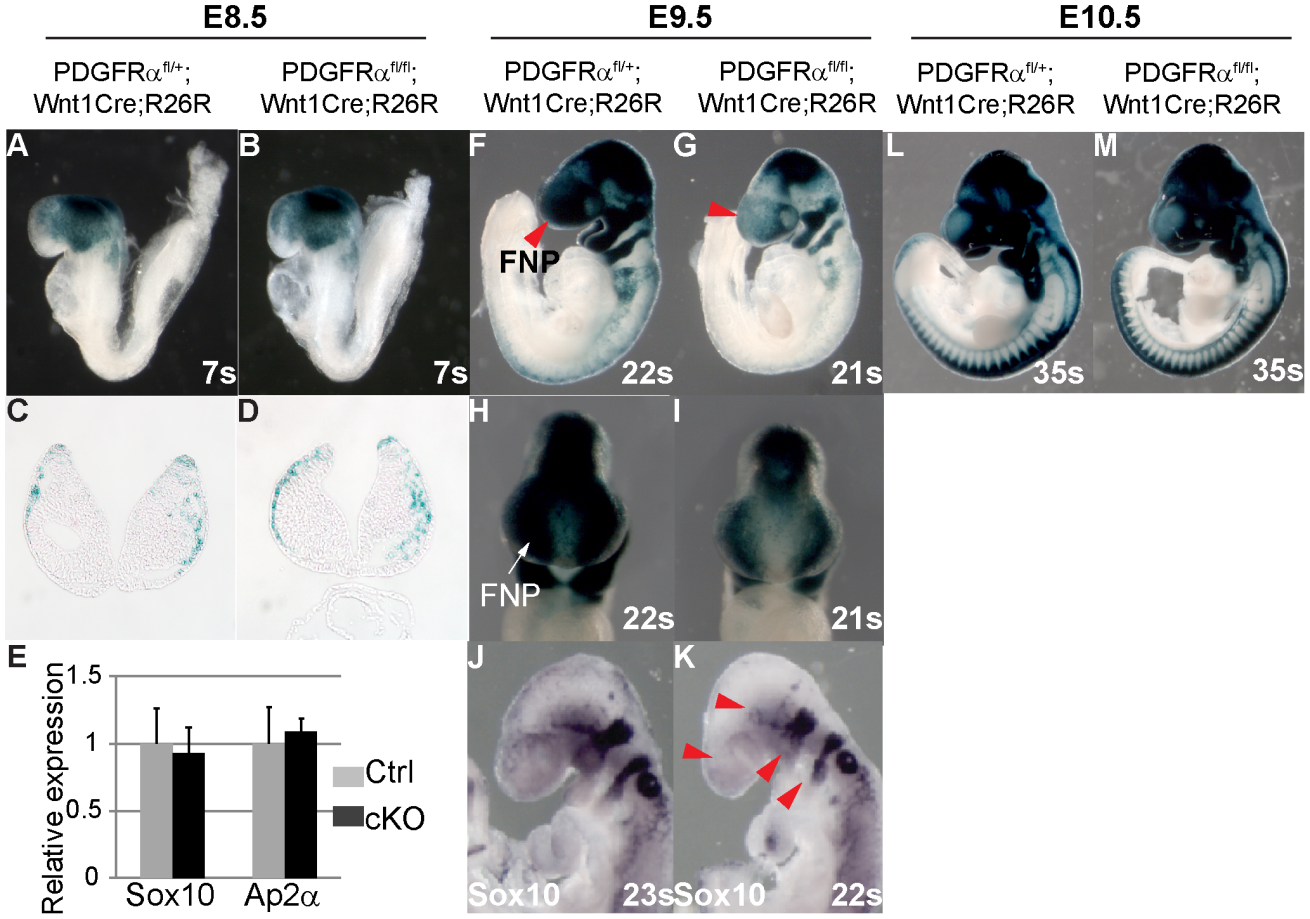
PDGFRα is required for proper population and migration of cranial neural crest cells. Embryos carrying the ROSA26R Cre reporter were examined by X-gal staining to highlight neural crest derived tissues. (A, B) Whole mount of E8.5 embryos. PDGFRα^fl/+^; Wnt1Cre (Ctrl) (A) and PDGFRα^fl/fl^; Wnt1Cre (cKO) (B) show comparable neural crest derived tissues. (C, D) Coronal sections of embryos through head mesenchyme. (E) qRT-PCR quantification of Sox10 and Ap2α mRNA expression of control and cKO embryos at E8.5. N = 3; asterisk, p>0.05. (F, G) CKO embryos (G) at E9.5 show fewer neural crest derived cells than control (F) in multiple tissues including frontal nasal process (FNP) (arrow in G) and pharyngeal arches. (H, I) Frontal view of stained embryos further confirms a neural crest populating defect in cKO FNP. (J, K) In situ hybridization of Sox10 reveals disrupted NCC formation in FNP, trigeminal ganglia, and pharyngeal arches in E9.5 cKO embryo (arrowheads in K). (L, M) Whole mount of E10.5 embryos. FNP, frontonasal prominence.

Lineage studies have revealed that MNP cells are predominantly derived from NCCs [1], [2]. To be able to trace NCCs in the conditional mutants, we introduced the R26R Cre reporter allele [33] into the PDGFRα^fl/fl^; Wnt1Cre background. CKO and control embryos were age matched by counting the number of somites. Lineage tracing showed that at E8.5 LacZ reporter expression was comparable in cKO and control embryos (Fig. 5A–D, cKO n = 7, ctrl n = 11), indicating that PDGFRα is not critical for neural crest specification. This observation was confirmed by quantifying the expression of neural crest marker genes Sox10 and Ap2α in E8.5 embryos using RT q-PCR (Fig. 5E, n = 3). By E9.5 however, R26R expression was attenuated in the cKO FNP as compared to the control (Fig. 5F–I, cKO n = 4, ctrl n = 15). Consistent with the above observations, Sox10 expression was also disrupted in E9.5 cKO embryos (Fig. 5J, K). At E10.5, cKO embryos showed fewer NCCs in pharyngeal arches III to VI (Fig. 5L, M, cKO n = 5, ctrl n = 7) and abnormal bifurcation of the NCCs streams migrating to these pharyngeal arches was observed in some embryos (Fig. S4A, B). Skeletal elements derived from these structures including the hyoid bone, the stapes, and the styloid process were severely deformed or missing in cKO embryos at E18.5 (Fig. S4C–H, n = 6). The cell lineage tracing results, along with the altered gene expression signature and defects in skeletal development indicate that PDGFRα is essential for NCCs to migrate in normal numbers and populate craniofacial regions.

### Defective migration and morphology of cKO NCCs

**Figure 6:**
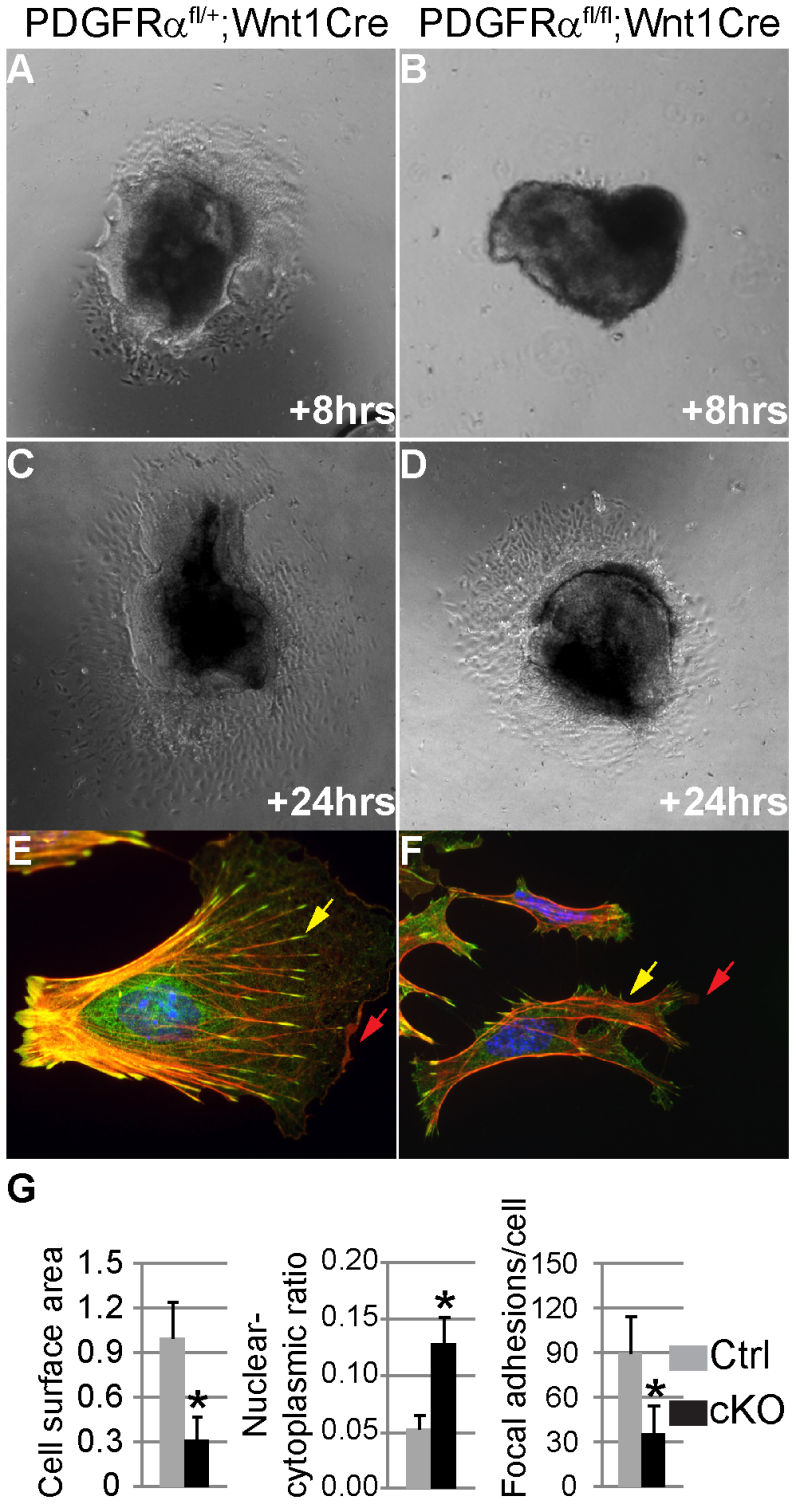
Altered NCC morphology in PDGFRα mutants. (A–D) Explant cultures of NCC. After 8 and 24 hour growth in 0.5% FBS and 10 µg/ml mitomycin C, cells emigrating from cKO (PDGFRα^fl/fl^; Wnt1Cre) neural crest explants (B, D) cover a smaller area than those from control (PDGFRα^fl/+^; Wnt1Cre) (A, C). (E, F) Immunostaining of emigrating NCCs of explants culture. F-actin was stained with Rhodamine-phalloidin (red), vinculin was stained with anti-vinculin antibody (green) and nuclei were stained with DAPI (blue). (E) A typical migrating NCC from control explants. (F) cKO NCCs show aberrant morphology, malformed lamellipodia (red arrows) and focal adhesions (yellow arrows). (G) Morphometric analysis of NCCs from control and cKO explants culture using Image J software. CKO NCCs exhibit smaller cell size, increased nuclear-cytoplasmic ratio and fewer focal adhesions (n = 50; asterisk, p<0.01).

To analyze the function of PDGFRα in NCC migration, explant cultures were established from the cranial neural tube (anterior to the first pharyngeal arch) of cKO and control embryos. To uncouple potential cell migration defects from alterations in cell proliferation, explants were plated on fibronectin in the presence of mitomycin C. The explant cultures exhibited a significant decrease in emigration of cKO NCC cells (Fig. 6A–D, n = 3). Moreover morphometric analysis indicated that primary cKO NCC cells were significantly smaller (33.1%) than control cells (n = 50), and exhibited fewer lamellipodia (Fig. 6E, F), which are required for cell migration. The cKO NCCs also exhibited an increased nuclear-cytoplasmic ratio (12.7% in cKO cells and 4.3% in wild type cells), as well as fewer focal adhesions (34.5 per cKO cell and 89.1 per wild type cell, n = 50; Fig. 6 G).

**Figure 7:**
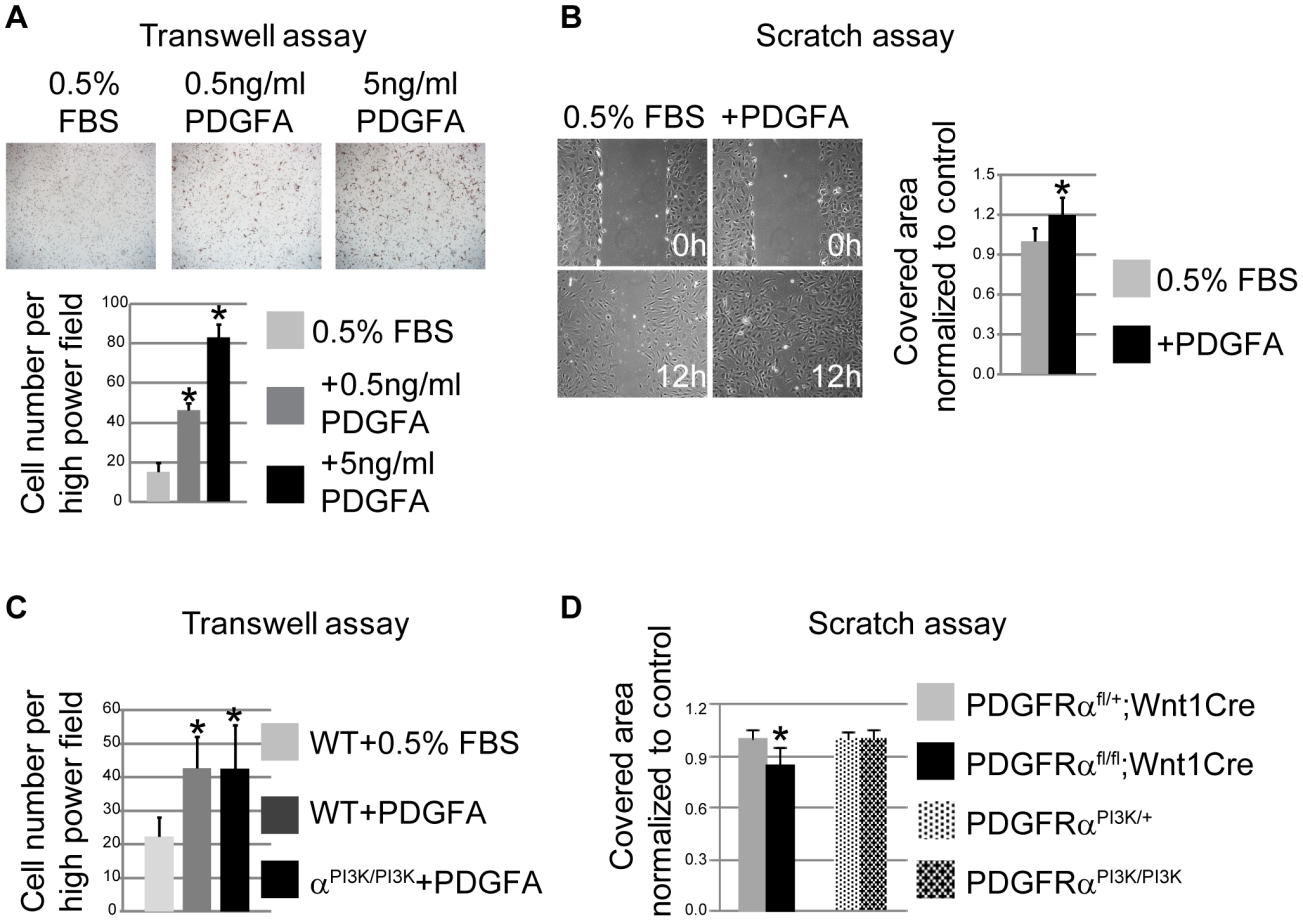
PDGFRα controls chemotaxis and cell motility. (A) PDGFA (0.5 ng/ml and 5 ng/ml) stimulates chemotaxis of MEPMs in transwell assays. N = 9; asterisk, p<0.01. (B) PDGFA (30 ng/ml) accelerates wound healing in MEPMs in scratch assays. N = 9; asterisk, p<0.01. (C) PDGFRα^PI3K/PI3K^ MEPMs migrate towards PDGFA at a comparable level to PDGFRα^PI3K/+^ MEPMs in transwell assays. N = 9; asterisk, p<0.01. (D) The speed of wound healing in scratch assays is significantly affected in cKO MEPMs, but not in PDGFRα^PI3K/PI3K^ MEPMs. N = 9; asterisk, p<0.01.

These results suggest that PDGFRα is essential for neural crest cell motility, possibly by regulating cytoskeletal architecture. Alternatively, PDGFA/PDGFRα might also regulate NCC migration by regulating cell guidance [25], [34]. To distinguish between these possibilities, we carried out further experiments in primary MEPMs at passage 1. PDGFA acted as a chemo-attractant of primary MEPMs in transwell assays (Fig. 7A). In addition, PDGFA treatment sped up the wound healing rate of MEPMs (Fig. 7B). These data indicate that activation of PDGFRα plays dual roles in neural crest derived cell migration, by stimulating chemotaxis and by regulating cell motility.

To investigate if PI3K signaling plays a role in NCC migration, we generated PDGFR^PI3K/PI3K^; Wnt1Cre; R26R^+/−^ embryos. Lineage tracing showed no obvious cNCC migration defects in PDGFRα^PI3K/PI3K^; Wnt1Cre; R26R^+/−^ embryos (Fig. S5, n = 6). Transwell assays revealed that PDGFRα^PI3K/PI3K^ MEPMs respond and migrate towards a source of PDGFA at a level comparable to wild type cells (Fig. 7C). In addition, the wound healing speed of PDGFRα^PI3K/PI3K^ MEPMs remains comparable to that of heterozygous cells in a scratch assay (Fig. 7D). In summary, these results indicate that PI3K signaling engaged by PDGFRα is not essential for cell migration, in contrast to its role in regulating proliferation of neural crest derived cells during development.

### Rac1 signaling mediates PDGFRα regulation of proliferation and migration of MEPMs

**Figure 8:**
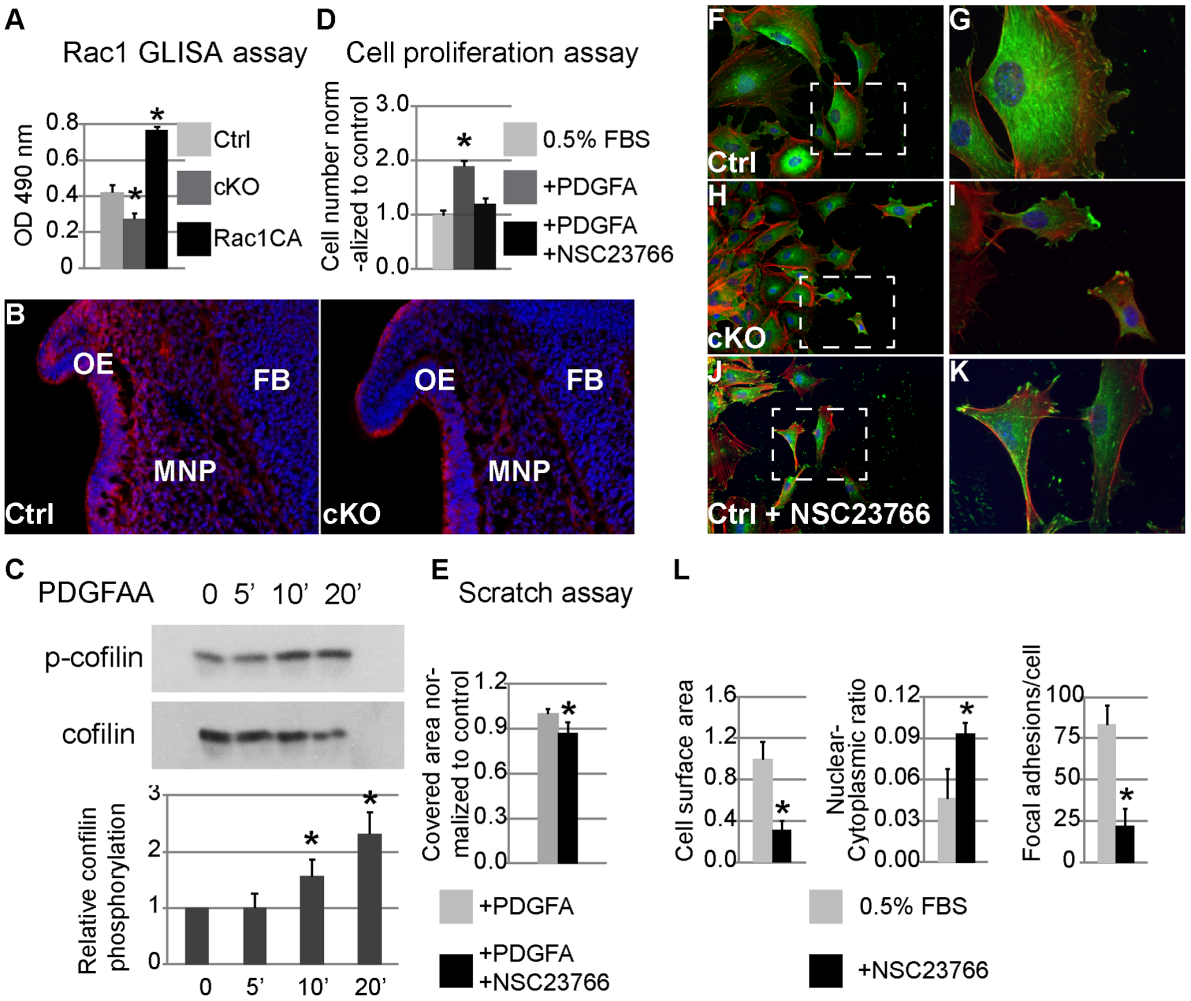
Rac1 mediates PDGFRα regulation of proliferation and migration in MNP cells and MEPMs. (A) Rac1 G-LISA assay. Rac1 activity is decreased in cKO MNP lysate; recombinant constitutively active (CA) Rac1 is included as a control. (B) Cofilin phosphorylation as seen by immunostaining (red) is attenuated primarily in cKO MNP mesenchymal cells (DAPI blue) (n = 3; asterisk, p<0.05). (C) Western blot analysis shows elevated cofilin phosphorylation in MEPMs following exposure to PDGFAA for 5 and 10 min. N = 3; asterisk, p<0.05. (D) Inhibition of Rac1 activity abrogates PDGFA stimulation of cell proliferation in MEPMs. N = 3; asterisk, p<0.05. (E) Inhibition of Rac1 activity antagonizes the effect of PDGFA on wound healing in MEPMs. N = 9; asterisk, p<0.01. (F–K) Immunostaining of MEPMs at the leading edge of scratch assay. Expression of Vinculin (green), F-actin (red) and nuclei (blue) was examined. G, I and K represent magnification of areas outlined by the dashed lines in F, H and J, respectively. (L) Compared to untreated wild type cells (F, G), inhibition of Rac1 leads to smaller cells, abnormal elongation and disrupted lamellipodia formation (J, K), similar to PDGFRα^fl/fl^; Wnt1Cre MEPMs (H, I). N = 50; asterisk, p<0.01.

The abnormal morphology of cKO NCCs indicates other signaling might be essential to regulate the cytoskeleton downstream of PDGFRα. Rho GTPases constitute a group of major regulators of cell migration that mediate actin reorganization, and lamellipodia and filopodia formation [35], [36]. PDGF and PI3K/Akt signaling have been shown to phosphorylate guanine nucleotide-exchange factors (GEFs), which in turn prompt the formation of GTP-bound, active small GTPases such as RhoA, Cdc42 and Rac1 [37]–[40]. Inactivation of Rac1 in NCCs caused facial clefting, strikingly resembling the PDGFRα cKO phenotype [41], [42], suggesting Rac1 to be a potential mediator of PDGFRα in NCC development. Rac1 activity was attenuated in lysates of E10.5 cKO MNP cells (Fig. 8A), and we observed a reduction in the expression of phosphorylated cofilin 1, an actin depolymerization enzyme required for cNCC development (Fig. 8 B) [43]. PDGFA stimulation facilitates phosphorylation of cofilin in MEPMs (Fig. 8C), indicating that PDGFRα can regulate Rac1 activity in neural crest derived cells. Consistent with an important role for Rac1 in mediating PDGF driven functions, treatment of MEPMs with the Rac1 specific inhibitor NSC 23766 blocked PDGFA stimulated proliferation and wound healing (Fig. 8D, E). Further examination revealed that inactivation of Rac1 affected lamellipodia formation at the leading edge of migrating MEPMs, reminiscent of the phenotype of PDGFRα deficient MEPMs (Fig. 8F–K). Inhibition of Rac1 activity in MEPMs also led to smaller size (31% of untreated cells, n = 50), fewer focal adhesion complexes (22.1 per treated cell vs. 83.2 per untreated cell, n = 50), and increased nuclear-cytoplasmic ratio (9% in treated cells vs. 5% in untreated MEPMs, n = 50) (Fig. 8L). Taken together, these results indicate a prominent role for Rac1 in the regulation of PDGF-induced cell migration and proliferation.

## Discussion

Facial clefting is a rare birth defect and its etiology remains poorly understood. In this work, we show that the facial clefting phenotype of PDGFRα mutants is not associated with a defect in NCC specification but rather a subsequent defect in the medial nasal process (MNP), a facial primordium derived from the frontonasal prominence (FNP). We further show that this defect is associated with alterations in both cell proliferation and cell migration, and that PI3K and Rac1 signaling are essential to maintain a normal level of cell proliferation. Last, we provide evidence that Rac1 regulates cell migration at the level of cell motility as well as chemotaxis under the regulation of PDGFRα.

A previous study from our laboratory had shown that the facial clefting observed in PDGFRα mutants was of neural crest origin, using chimeric analysis and conditional mutagenesis with the Wnt1Cre driver [21]. Although global defects in cell proliferation and migration were not documented, chimeric analysis identified a role for PDGFRα in development of the pharyngeal arches, which we now show by lineage tracing are deficient in cNCCs by E10.5 in cKO embryos. In this work, we have considerably refined the analysis by examining specific rather than overall craniofacial subregions. We were thus able to document disruption of cell migration in the FNP by cell lineage analysis at E9.5, and of cell proliferation in the MNP of cKO embryos by E10.5. Therefore, both the previous study and the present work identify a crucial role for PDGFRα in NCC development.

We found that PDGFRα exhibits a strong expression pattern in the MNP mesenchyme at different stages. PDGFA is expressed in the MNP epithelium, and PDGFC is expressed in both the MNP epithelium and the mesenchyme, consistent with paracrine or autocrine PDGFRα signaling during craniofacial development. Prior genetic evidence from our lab, using point mutations in the PI3K binding sites in the PDGFRs, has implicated PI3K as the key signaling pathway regulating craniofacial development [24]. We also found that that PI3K signaling regulates p44/42 MAPK (data not shown). Strikingly, mice carrying a neural crest-specific deletion of Erk1/Erk2 display facial clefting [23]. p44/42 MAPK can also be engaged by other pathways than PI3K, and by other RTKs that are critical for craniofacial development including Fgfrs, Eph receptors, EGFR, Ror or Ryk. Although these RTKs share some similar intracellular domains and engage overlapping signaling pathways, their impact on craniofacial development might reflect tissue-restricted expression patterns of receptors and ligands, as well as engagement of unique combinations of downstream signaling cascades. It will be important to understand if the phenotypic differences associated with different RTKs are due to dosage variation of PI3K signaling, involvement of other unique signaling pathways, or a combination of both.

Neural crest cells form through delamination of cells at the lateral plate border of the neural tube that undergo an epithelial to mesenchymal transition. There is extensive evidence that cell-cell contacts through intricate lamellipodial and filipodial extensions play critical roles in regulating how cells exit the neural tube and migrate to their proper destination (for a review, see [13]). Small GTPases, including Rho, Rac and Cdc42 are well known to regulate such cell behaviors, but also cell proliferation and gene transcription under the regulation of multiple RTKs [44]. Recent gene targeting studies showed that inactivation of Rac1 or Cdc42, or overexpression of a dominant negative Rho kinase in mouse NCCs causes a severe facial clefting phenotype, which strikingly resembles PDGFRα homozygous mutant embryos [21], [22], [41], [42], [45]. In particular, Rac1 deficient NCCs exhibit decreased proliferation, abnormal cell morphology, as well as disrupted lamellipodia formation [42], very similar to the defects we have observed in PDGFRα deficient NCCs and MEPMs. The notion that PDGFRα might be a major regulator of Rac1 activity is further supported by our present work and other studies that show that PDGFA stimulates Rac1-GTP levels in a variety of biological settings [46]–[48]. It has been suggested that EGFR could be the major effector of the Rac1 mutant phenotype [41], as EGF has been used as an agonist of Rac1 activity in a variety of *in vitro* studies. However EGFR null mutant mice exhibit a much milder craniofacial phenotype with only a cleft palate. In addition, gene expression data showed that EGFR and Rac1 only partially overlap in ectodermal cells during early stages of craniofacial morphogenesis (Emage, www.emouseatlas.org), whereas PDGFRα shows a broad expression pattern similar to Rac1 in cNCCs at E10.5. Functionally, PDGFRα deficient NCCs exhibit abnormal morphology and defective formation of lamellipodia and focal adhesions. These lines of evidence point to a major role for Rac1 in mediating PDGFRα functions in NCC development and craniofacial morphogenesis.

## Materials and Methods

### Ethics statement

The Mount Sinai School of Medicine Institutional Animal Care and Use Committee (IACUC) approved all animal work and procedures used in this study. The Mount Sinai animal facility is accredited by the Association for Assessment and Accreditation of Laboratory Animal Care International (AAALAC).

### Mice and genotyping

PDGFRα^fl/fl^, PDGFRα^GFP/+^, PDGFRα^PI3K/+^, Wnt1Cre and R26R mice have been described previously [21], [24], [26], [29], [33]. PDGFRα^fl/fl^, PDGFRα^PI3K/+^ and Wnt1Cre mice were maintained on a 129S4 co-isogenic genetic background, and PDGFRα^GFP/+^ and ROSA26R mice were kept on a C57BL/6J co-isogenic background. Mice and embryos used in lineage tracing studies were maintained on a mixed genetic background.

### Histology, skeletal analysis, immunostaining, in situ hybridization and X-gal staining

For histology, staged embryos were dissected in ice-cold PBS, fixed in Bouin's fixative, dehydrated through a graded series of ethanol washes, and embedded in paraffin. Sections were cut at 10 µm for hematoxylin and eosin staining. Skeletal analysis was performed on E18.5 embryos as described [22]. Craniofacial morphometry was performed as described in Fig. S6. For immunostaining, embryos were fixed in 4% PFA overnight at 4°C, dehydrated in 30% sucrose/PBS and embedded in OCT. Cryosections were prepared at a thickness of 10 µm. Immunostaining was performed according to standard protocols using antibodies to PDGFRα (1∶60; Santa Cruz), vinculin (1∶200, Sigma), BrdU (1∶500, DSHB), Cleaved-Caspase 3 (1∶200, Cell Signaling Technology) and rhodamine phalloidin (0.2 µM, Biotium). For in situ hybridization, embryos were dissected in ice-cold PBS, fixed in 4% paraformaldehyde (PFA), dehydrated through graded ethanol washes, and embedded in paraffin. Coronal sections were cut at 10 µm and in situ hybridization was performed as described [49]. X-gal staining was performed as described [33].

### Cell proliferation analysis

To measure cell proliferation rates in vivo, BrdU was injected intraperitoneally into pregnant females at a dosage of 50 µg per gram of body weight. Embryos were dissected after 1 hour, fixed in 4% PFA and processed for cryosections and immunostaining using standard protocols. BrdU labeled cells were counted in a random area in the defined mesenchyme at comparable levels in mutant and control samples. Three continuous sections were counted from each of triplicate samples. The result of BrdU labeling was presented as percentage of BrdU-positive cells against total nuclei labeled by DAPI. Student's *t*-test was used to determine statistical significance.

### Neural crest explant cultures

Neural tubes anterior to the first pharyngeal arch were dissected from E8.5 embryos. Following two brief washes in ice-cold PBS, heads were sagitally split into two equal halves. The tissue was incubated in 0.5% trypsin/2.5% pancreatin in PBS for 5 minutes on ice, and then in DMEM with 10% FBS for 10 minutes to stop the reaction. The head mesenchyme was carefully dissected and isolated with fine-tipped Dumont #5 tweezers, and the neural tube was transferred to fibronectin-coated cover slips in a 6 well plate. For NCC emigration assays, neural tubes were cultured in DMEM/F12 with 10% FBS for 6 hours. The explants were then treated with low serum media (DMEM/F12 containing 0.5% FBS) with 10 µg/ml mitomycin C for two hours. The culture medium was replaced with low serum media and maintained for 24 hours. Results were recorded at 8 hours and 24 hours respectively. The explants were then removed and the emigrating cells were subjected to immunostaining.

### Cell culture, scratch assay and transwell assay

Primary Mouse Embryonic Palatal Mesenchymal cells (MEPMs) were isolated as described [50]. For scratch assays, MEPMs at passage 1 were seeded on fibronectin-coated cover slips in 6 well plates at a density of 100,000 cells per well. After reaching 70–80% confluency, cells were starved for 24 hours in DMEM containing 0.5% FBS. In some experiments, serum starved cells were pretreated with 30 µM Rac1 inhibitor NSC23766 (R&D Systems) for 3 hrs before stimulation with 30 ng/ml PDGFA. The scratch was mechanically created using a sterile P200 pipette tip and washed twice with starving medium to remove cell debris. The wound area was then photographed at marked positions (3 different fields per well). Cells were allowed to migrate for 12 hours at 37°C before the same fields were recorded. All experiments were performed in triplicate. Scratch results were measured with Image J software (NIH, Bethesda, USA) and analyzed using the extension package MiToBo [51].

For transwell assays, cell culture inserts for a 24-well plate (Fisher) with a pore size of 8 µm were coated with fibronectin. P1 MEPMs were trypsinized, washed, and suspended in serum free medium at a concentration of 5×10^6^ cells/ml. 300 µl of cell suspension was added to the insert chambers immersed in 500 µl medium with 10% FBS, 0.5% FBS, or 0.5% FBS with PDGFAA. After incubation at 37°C and 5% CO2 for 3.5 hours, the inserts were fixed in 3.7% formaldehyde and stained in Mayer's hematoxylin solution. Filters of the inserts were then isolated with a scalpel and mounted. The numbers of cells on the bottom were counted. Data were recorded from 9 high power fields from three independent experiments.

### Western blot and Rac1 activity analysis

Western blot analysis was carried out as described previously [24], using primary antibodies from Abcam: anti-cofilin (phospho-S3) and from Cell Signaling Technology: anti-cofilin, anti-p44/42 MAPK, anti-phospho p44/42 MAPK, anti-Akt, anti-phospho-Akt (Ser473). Chemical inhibitors for MAPK (U0126, Promega), PI3K/Akt (LY294002, Stemgent) and Rac1 (NSC23766, R&D systems) were used in lysate generation for western blot analysis and the Rac1 activity assay, following the manufacturers' instruction. Rac1 activity analysis was performed using the colorimetric based G-LISA Rac1 Activation Assay Biochem Kit (Cytoskeleton Inc.).

## Supporting Information

Figure S1 Section in situ hybridization shows unaltered Alx4 and Six1 expression in PDGFRα cKO embryos at E10.5. (A) In control embryos, Six1 mRNA is expressed in MNP mesenchyme and OE at anterior level. At posterior level, Six1 expression is detected in MX and MD mesenchyme. Six1 expression level and pattern are not affected in PDGFRα cKO embryos. (B) Alx4 mRNA is detected in LNP and MNP mesenchyme in the control embryos, at a higher level in the anterior LNP. At the posterior level, Alx4 expression is detected in the dorsal region of MX and medial portion of MD mesenchyme. Alx4 expression is comparable in PDGFRα cKO and control embryos. LNP, lateral nasal process; MNP, medial nasal process; OE, olfactory epithelium; MD, mandible; MX, maxilla.
(TIF)

Figure S2 CNCC derived MEPMs express PDGFRα and respond to PDGFA stimulation. (A) MEPMs prepared from PDGFRα^GFP/+^ embryos express PDGFRα, at both mRNA (shown by GFP expression) and protein (shown by immunostaining with anti- PDGFRα antibody) levels. (B) MEPMs respond to PDGFA stimulation and exhibit increased phosphorylation of Akt and p44/42 MAPK, both known to be downstream of PDGFRα signaling.
(TIF)

Figure S3 PDGFA treatment fails to stimulate cell proliferation of PDGFRα^PI3K/PI3K^ MEPMs. Cell proliferation assay reveals that PDGFA stimulation (30 ng/ml) promotes cell proliferation in wild type (WT) MEPMs, but fails to do so in PDGFRα^PI3K/PI3K^ MEPMs. N = 3; asterisk, p<0.05.
(TIF)

Figure S4 PDGFRα is required for normal development of pharyngeal arches and their skeletal derivatives. (A) In E10.5 control embryos, neural crest cells migrate in collective streams and populate the pharyngeal arches (labeled as I–VI). (B) cKO embryos exhibit hypoplasia of pharyngeal arches (arrow) and abnormal bifurcation of streams of neural crest cells (arrowheads). (C,D) Lateral view of E18.5 PDGFRα^fl/+^; Wnt1Cre and PDGFRα^fl/fl^; Wnt1Cre skeletal preparations. (E, F) Detailed view of hyoid bones and cartilages of control and cKO embryos at E18.5. (G, H) Detailed view of middle ear bones and adjacent NC derived structures in control and cKO embryos. Roman numerals represent different pharyngeal arches. Asterisks refer to deformed bones or cartilages in cKO. bh, body of hyoid bone; cr, cricoid cartilage; g, greater horn; l, lesser horn; in, incus; ma, maleus; MC, Meckel's cartilage; rtp, retrotympanic process; sp, styloid process; sq, squamous bone; st, stapes, th, thyroid cartilage; tr, trachea rings.
(TIF)

Figure S5 PDGFRα^PI3K/PI3K^ embryos show normal cNCC development at E9.5 and E10.5. (A–D) NCC lineage tracing results in PDGFRα^PI3K/+^; Wnt1Cre; R26R^+/−^ and PDGFRα^PI3K/PI3K^; Wnt1Cre; R26R^+/−^ embryos at E9.5. N = 6. (A, B) Lateral view of whole mount embryos. (C, D) Frontal view of whole mount embryos. (E, F) Lateral view of pharyngeal arches at E10.5. N = 4.
(TIF)

Figure S6 Measurement and quantification of nasal cartilage and premaxilla in the prepared craniofacial skeleton at E18.5. (A) Illustration of measurement of nasal cartilage, premaxilla and the total length of the head in a ventral view of the mouse skull. a = length of nasal cartilage, b = length of premaxilla, and c = distance from nasal cartilage to supraoccipital bone. (B) The length index of nasal cartilage or premaxilla was calculated as the actual length of each (a or b) divided by the total skull length (c), and then normalized by the control. The result shows that cKO nasal cartilage and premaxilla are significantly shorter than the control. N = 6, asterisk: p<0.01. (C) PDGFRα^PI3K/PI3K^ embryos exhibit shorter nasal cartilage and premaxilla than their littermates PDGFRα^PI3K/+^. n = 5, asterisk: p<0.05. Als, alisphenoid; bs, basisphenoid; FB, forebrain; MD, mandible; MNP, medial nasal process; MX, maxilla; nc, nasal cartilage; NS, nasal septum; pmx, premaxilla; ptg, pterygoid; so, supraoccipital bone; T, tongue.
(TIF)
