# Supplementary figures and images for: A Critical Role for PDGFRα Signaling in Medial Nasal Process Development
Source: PLoS Genet. 2013 Sep 26;9(9):e1003851. doi: 10.1371/journal.pgen.1003851 (PMC3784569; doi:10.1371/journal.pgen.1003851)

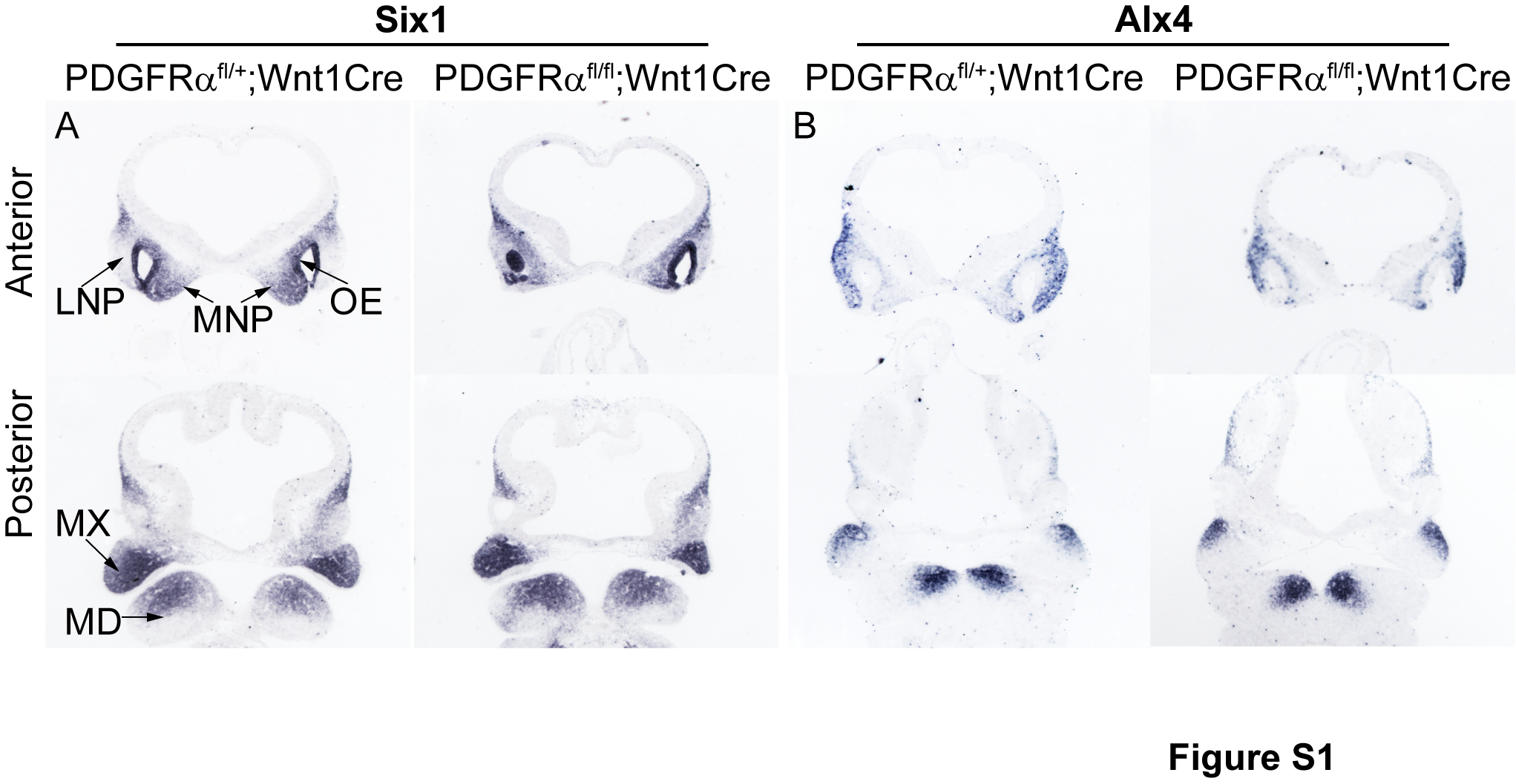

Supplement: Figure S1 — Section in situ hybridization shows unaltered Alx4 and Six1 expression in PDGFRα cKO embryos at E10.5. (A) In control embryos, Six1 mRNA is expressed in MNP mesenchyme and OE at anterior level. At posterior level, Six1 expression is detected in MX and MD mesenchyme. Six1 expression level and pattern are not affected in PDGFRα cKO embryos. (B) Alx4 mRNA is detected in LNP and MNP mesenchyme in the control embryos, at a higher level in the anterior LNP. At the posterior level, Alx4 expression is detected in the dorsal region of MX and medial portion of MD mesenchyme. Alx4 expression is comparable in PDGFRα cKO and control embryos. LNP, lateral nasal process; MNP, medial nasal process; OE, olfactory epithelium; MD, mandible; MX, maxilla. (TIF) [file pgen.1003851.s001.tif]

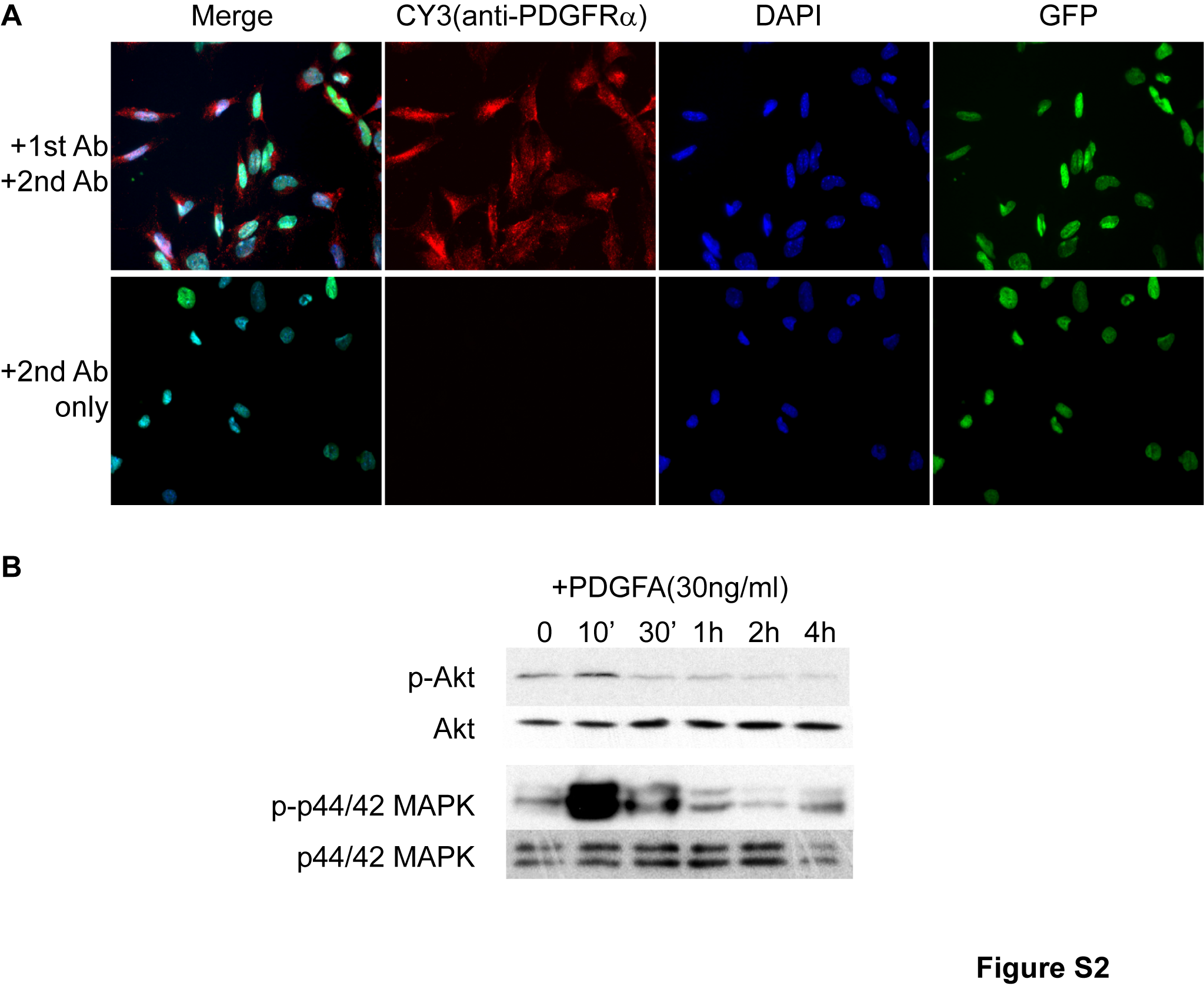

Supplement: Figure S2 — CNCC derived MEPMs express PDGFRα and respond to PDGFA stimulation. (A) MEPMs prepared from PDGFRαGFP/+ embryos express PDGFRα, at both mRNA (shown by GFP expression) and protein (shown by immunostaining with anti- PDGFRα antibody) levels. (B) MEPMs respond to PDGFA stimulation and exhibit increased phosphorylation of Akt and p44/42 MAPK, both known to be downstream of PDGFRα signaling. (TIF) [file pgen.1003851.s002.tif]

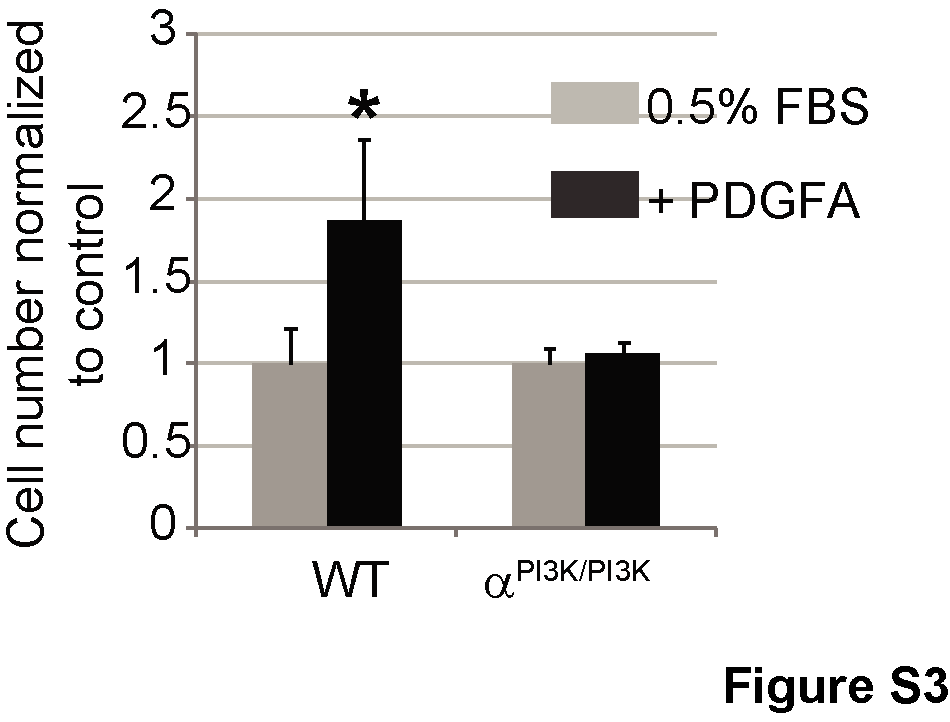

Supplement: Figure S3 — PDGFA treatment fails to stimulate cell proliferation of PDGFRαPI3K/PI3K MEPMs. Cell proliferation assay reveals that PDGFA stimulation (30 ng/ml) promotes cell proliferation in wild type (WT) MEPMs, but fails to do so in PDGFRαPI3K/PI3K MEPMs. N = 3; asterisk, p<0.05. (TIF) [file pgen.1003851.s003.tif]

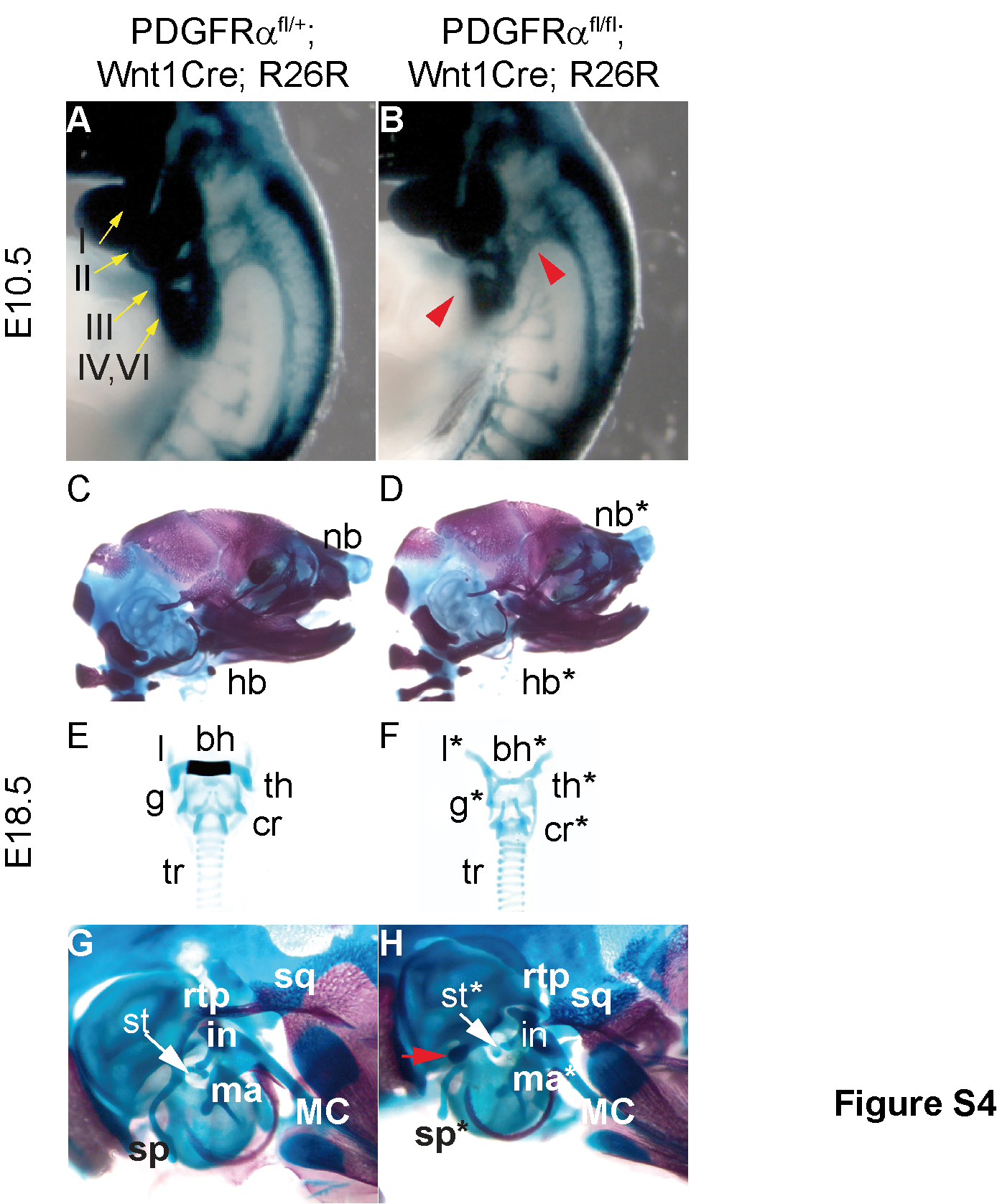

Supplement: Figure S4 — PDGFRα is required for normal development of pharyngeal arches and their skeletal derivatives. (A) In E10.5 control embryos, neural crest cells migrate in collective streams and populate the pharyngeal arches (labeled as I–VI). (B) cKO embryos exhibit hypoplasia of pharyngeal arches (arrow) and abnormal bifurcation of streams of neural crest cells (arrowheads). (C,D) Lateral view of E18.5 PDGFRαfl/+; Wnt1Cre and PDGFRαfl/fl; Wnt1Cre skeletal preparations. (E, F) Detailed view of hyoid bones and cartilages of control and cKO embryos at E18.5. (G, H) Detailed view of middle ear bones and adjacent NC derived structures in control and cKO embryos. Roman numerals represent different pharyngeal arches. Asterisks refer to deformed bones or cartilages in cKO. bh, body of hyoid bone; cr, cricoid cartilage; g, greater horn; l, lesser horn; in, incus; ma, maleus; MC, Meckel's cartilage; rtp, retrotympanic process; sp, styloid process; sq, squamous bone; st, stapes, th, thyroid cartilage; tr, trachea rings. (TIF) [file pgen.1003851.s004.tif]

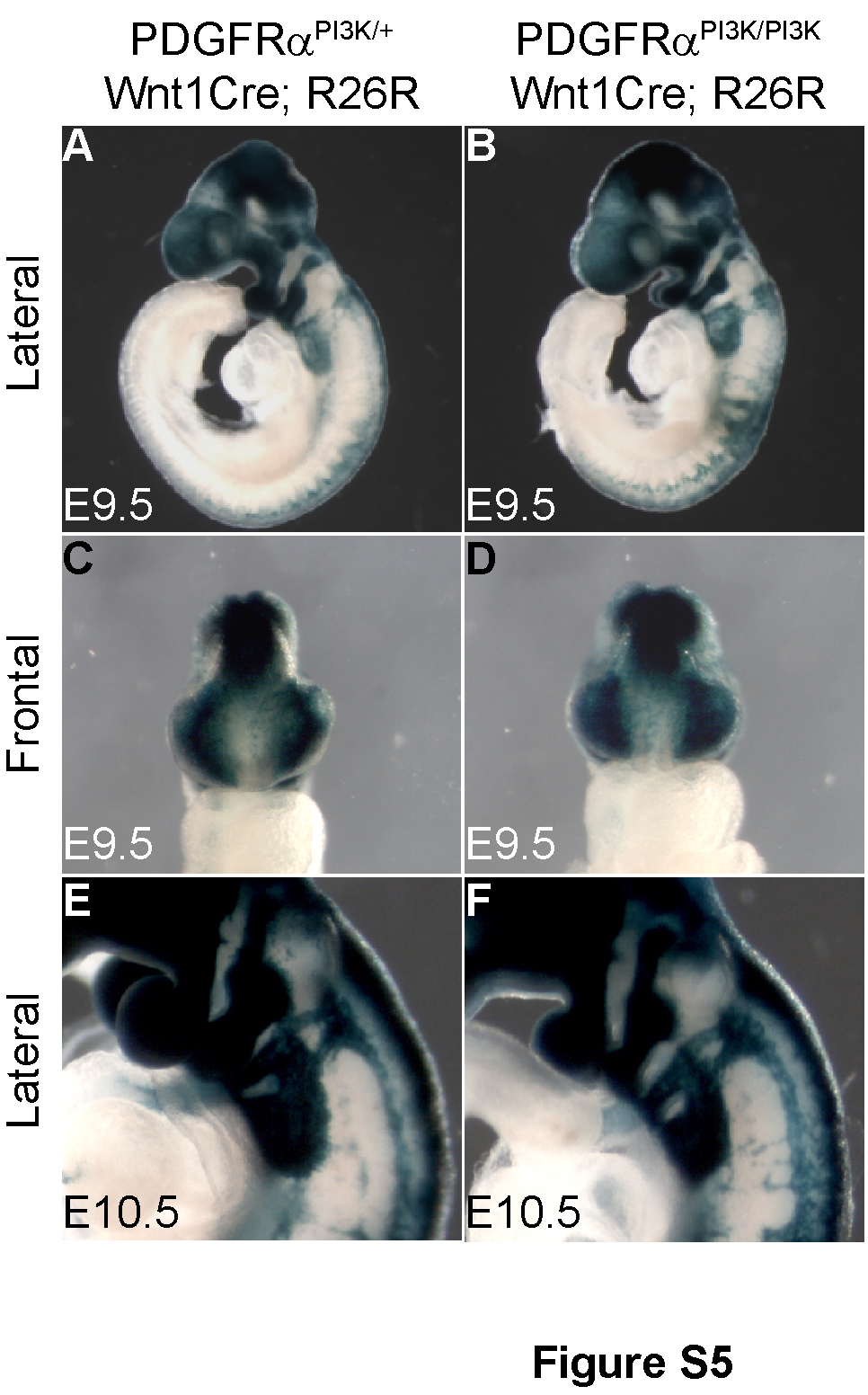

Supplement: Figure S5 — PDGFRαPI3K/PI3K embryos show normal cNCC development at E9.5 and E10.5. (A–D) NCC lineage tracing results in PDGFRαPI3K/+; Wnt1Cre; R26R+/− and PDGFRαPI3K/PI3K; Wnt1Cre; R26R+/− embryos at E9.5. N = 6. (A, B) Lateral view of whole mount embryos. (C, D) Frontal view of whole mount embryos. (E, F) Lateral view of pharyngeal arches at E10.5. N = 4. (TIF) [file pgen.1003851.s005.tif]

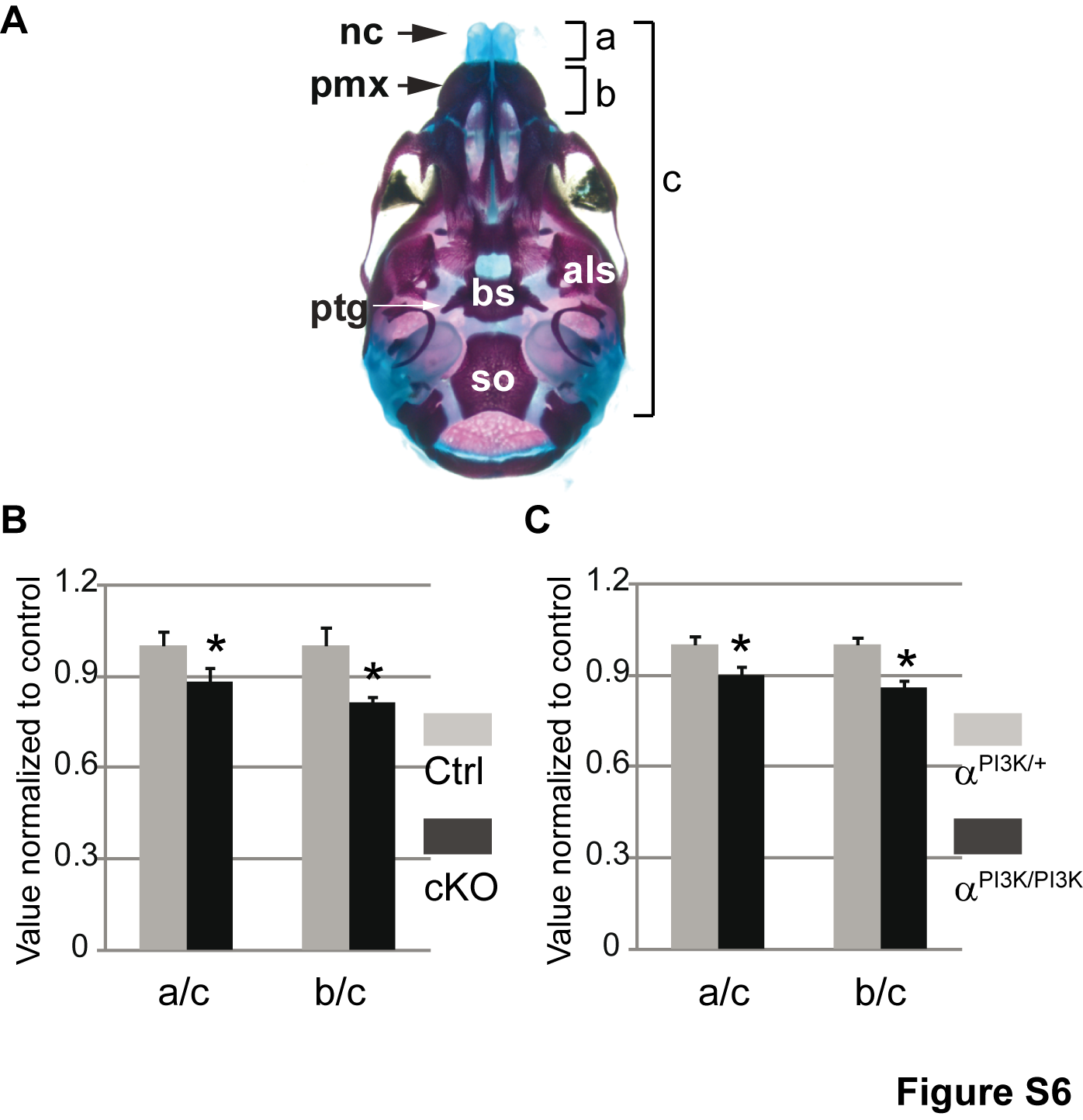

Supplement: Figure S6 — Measurement and quantification of nasal cartilage and premaxilla in the prepared craniofacial skeleton at E18.5. (A) Illustration of measurement of nasal cartilage, premaxilla and the total length of the head in a ventral view of the mouse skull. a = length of nasal cartilage, b = length of premaxilla, and c = distance from nasal cartilage to supraoccipital bone. (B) The length index of nasal cartilage or premaxilla was calculated as the actual length of each (a or b) divided by the total skull length (c), and then normalized by the control. The result shows that cKO nasal cartilage and premaxilla are significantly shorter than the control. N = 6, asterisk: p<0.01. (C) PDGFRαPI3K/PI3K embryos exhibit shorter nasal cartilage and premaxilla than their littermates PDGFRαPI3K/+. n = 5, asterisk: p<0.05. Als, alisphenoid; bs, basisphenoid; FB, forebrain; MD, mandible; MNP, medial nasal process; MX, maxilla; nc, nasal cartilage; NS, nasal septum; pmx, premaxilla; ptg, pterygoid; so, supraoccipital bone; T, tongue. (TIF) [file pgen.1003851.s006.tif]
